# Trisomy 18 and the possibility of choice: The importance of Perinatal Hospice’s support

**DOI:** 10.1007/s00431-025-05970-8

**Published:** 2025-01-16

**Authors:** D. Visconti, V. Esposito, F. Brugnoli, V. Gallitelli, B. Corsano, P. Papacci, M. Pellegrino, M. De Santis, A. Lanzone, G. Noia

**Affiliations:** 1 Unit of Obstetrics and Gynecology, Università Cattolica del Sacro Cuore, Largo Francesco Vito 1, Rome, Italy; 2 Department of Women and Child Health, Women Health Area, Fondazione Policlinico Universitario Agostino Gemelli IRCCS, Largo Gemelli 8, Rome, Italy; 3 Department of Healthcare Surveillance and Bioethics, Università Cattolica del Sacro Cuore, Rome, Italy; 4 Neonatal Intensive Care Unit, Fondazione Policlinico Universitario A. Gemelli IRCCS, Largo Gemelli 8, Rome, Italy

**Keywords:** Ethics, Neonatology, Obstetrics, Syndrome, Palliative, Genetics

## Abstract

Trisomy 18 is a severe aneuploidy associated with multiple malformations and a poor prognosis. The diagnosis is typically made prenatally, leading to a high rate of pregnancy terminations. The aim of this study is to demonstrate that even though the prognosis is heterogeneous, prolonged survival is possible and these children are an enrichment for their families after all. A retrospective, descriptive and monocentric study was conducted on fetuses diagnosed with trisomy 18, evaluated between March 2017 and June 2023 at our Institution. The objective was to investigate the natural history of trisomy 18 and the psychological impact on parents who choose to carry the pregnancy on, through a retrospective data collection and the use of a questionnaire. Sixteen couples with a diagnosis of trisomy 18 were cared for within the Perinatal Hospice Pathway during the study period. Cardiac defects were identified in 93.7% of cases, structural abnormalities in 71.4%, respiratory defects in 14.3% of the fetuses, while genitourinary defects affected nearly half of the study population. The survival rate was typically less than one day, however two babies survived for more than four years. All couples reported being satisfied with their decision to continue the pregnancy and would do so again if given the opportunity.

*Conclusions*: despite the severity of the diagnosis, couples may choose to continue the pregnancy and give birth. Our study shows that trisomy 18 is not merely a lethal condition and the Perinatal Hospice plays a crucial role in supporting these families.

**What is Known:**

*• Trisomy 18 is a severe aneuploidy with a poor prognosis and a high rate of pregnancy terminations.*

**What is New:**

*• Despite the severity of this condition, prolonged survival is a possibility. If couples are adequately supported by Perinatal Hospices, they may choose to continue the pregnancy, thereby enriching their experience.*

## Introduction

Trisomy 18 was first described by Edwards et al. in. 1960 [1]. It is an autosomal chromosomal disorder and the second most common aneuploidy after trisomy 21 [2, 3]. The prevalence is 1 in 2.500 [3] but the live birth prevalence ranges from 1 in 3.600 to 1 in 10.000 because of the high rate of pregnancy terminations and stillbirths [2, 4]. The prevalence has increased over the last 20 years because of the advanced maternal age in many pregnant women [3, 5]. Among live born, it is more common in females than males (3:1) [2, 5]. Trisomy 18 is a severe condition and its main clinical features include cardiovascular, musculoskeletal, nervous, digestive and renal malformations, orofacial clefts and omphalocele [6, 7]. The majority of Trisomy 18 syndromes are diagnosed prenatally: screening tests can detect a high risk for aneuploidy but the final diagnosis requires a diagnostic test, like an amniocentesis or a chorionic villous sampling [2, 4]. The prognosis is poor: 70% of pregnancies spontaneously interrupt [8], the rate of preterm delivery is high and 40% of fetuses don’t survive labor [2]. About 13,5% of fetuses are live born and their median survival time goes from 3 to 14,5 days [2, 7]. For a long time, trisomy 18 has been considered a lethal condition, not compatible with life, therefore, no treatments were provided to children and many couples interrupted the pregnancy. Nowadays many couples continue the pregnancy and the scenario has changed since specific treatments are provided to patients, both therapeutic and palliative [9]. Clinical experience shows that, if families are offered multidisciplinary support, then they may choose to carry the pregnancy on, despite the pressures to interrupt it that often come from the clinical setting [10, 11]. The aim of this study is to present the cases of trisomy 18 who had access to the Perinatal Palliative Care Center S. Mother Teresa of Calcutta (Fondazione Policlinico Universitario Agostino Gemelli IRCCS, Roma) from 2016 to 2023 to show that, despite the severity of this condition, the prognosis is heterogenous, the outcome is not always predictable and those children are an enrichment for their families after all.

## Materials and methods

We carried out a retrospective, descriptive and monocentric study of fetuses with a prenatal diagnosis of trisomy 18 evaluated at our Institution between March 2017 and June 2023. The study was approved by the internal Ethic Committee Board (N. protocol DIPUSVSP-22–02–231). All couples enrolled gave informed consent for the study. Couples were referred to our Perinatal Hospice by various italian prenatal diagnosis centers and during the first meeting had a consultation with our entire team of specialists. Our Perinatal Hospice is supported by “Il Cuore in una Goccia”, a non-profit foundation that provides families with psychological, spiritual and economic support. Moreover, the Foundation aims to create a network in which families who experienced the loss of a baby under similar circumstances can support other families going through the same experience. The aim of the study was to explore the natural history of trisomy 18 and the psychological impact on parents who decide to continue the pregnancy. Data were collected from the hospital informatic systems: in particular, we collected data about maternal demographic characteristics, ultrasonographic prenatal findings, fetal/neonatal outcome and mode of delivery. At the end of the data collection a questionnaire was administered to the families, by phone or by e-mail, in order to highlight some psychological aspects related to this experience. The questionnaire gathered the following open-ended questions:

- “Please tell us the reason why you chose to continue the pregnancy.”
- “Did your partner and you agree with the decision to continue the pregnancy?”
- “Would you make the same choice again?”
- “Have you had any other pregnancies after this event?”

## Results

### Demographic information

Sixteen couples were cared for by our Perinatal Hospice during the study period. All couples were included in a codified clinical-care pathway. During the prenatal phase, they underwent regular check-ups in an obstetric outpatient clinic, with access to all necessary specialist consultations depending on the anomalies detected during ultrasound examinations (genetics, pediatric cardiology and cardiac surgery, child neuropsychiatry, pediatric neurosurgery, pediatric nephrology, pediatric surgery). Throughout the Perinatal Hospice pathway couples were supported by:

1. Regular psychological consultations;
2. Spiritual support, if desired;
3. Accompaniment by a family from the "Il Cuore in una Goccia" foundation, who had previously experienced a similar situation.

For each couple, the Perinatal Hospice team held one or more multidisciplinary meetings, always involving the clinical ethics consultation. Following each interdisciplinary evaluation, the team shared the identified clinical-ethical care orientation with the couple. Based on this discussion, the ethics consultant developed a Shared Document [12] that outlined the care plan for key moments of the pregnancy, including potential prenatal palliative treatments, the type and timing of delivery, assistance at birth, and the possibility of Baptism in the delivery room. This Shared Document was signed by both the Perinatal Hospice team and the parents and was included in the pregnant woman’s medical record.

**Fig. 1:**
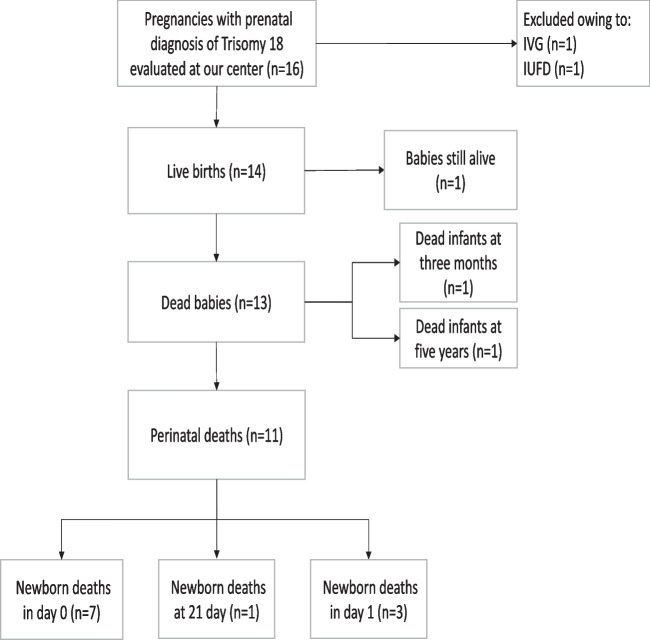
Flowchart summarizing inclusion of pregnancies with prenatal diagnosis of Trisomy 18. *IVG (voluntary interruption of pregnancy); IUFD (intrauterine fetal demise)*

Fourteen babies were born alive, one pregnancy resulted in fetal demise and one couple interrupted the pregnancy. Among liveborn babies, 7 died within few hours; 3 died within the first day; one died at 21 days; one died at three months; one reached the age of 5 and finally one baby girl is still alive (Fig. 1).

The liveborn babies received Baptism at birth, if the parents wished so, along with photos and videos as keepsakes. Both parents were always present, including in cases of cesarean delivery. Babies who died within a few hours were provided with comfort care. Those who survived were reassessed by the Perinatal Hospice team, involving the clinical specialists consulted prenatally and the ethics consultation to evaluate the proportionality of intensive or invasive treatments.

As showed in Table 1, the mean maternal age was 38.3 years (29–45). The type of delivery was cesarean section in 10 (71,4%) cases and vaginal delivery in 4 (28.6%) cases. The mean gestational age at delivery was 35,5 weeks (range, 32–37) and the mean neonatal weight was 1456,5 gr (range 1050–2135 gr). Eleven (78,6%) babies were females, 3 (21,4%) were males*.* The mean gestational age at diagnosis was 18,7 (range, 13–31) weeks. Nine (56,2%) couples did a non-invasive prenatal test and they all resulted in a high risk for aneuploidies. 13 (81,2%) patients underwent an invasive procedure for the final diagnosis. Three (18.7%) couples chose not to undergo invasive procedures.

**Table 1:** Baseline characteristics of the initial 16 studied pregnancies with prenatal diagnosis of Trisomy 18. Variables are reported as mean (range) and n (%)

*Characteristics*	*All cases n* = *16*
Maternal age (*year*)	38.3 (29–45)
Gestational age at diagnosis (*week*)	18.7 (13–31)
Diagnostic invasive procedures Amniocentesis Chorionic villous sampling Cystocentesis	13/16 (81.2) 9/13 (69.2) 3/13 (23) 1/13 (7.7)
Delivery Vaginal delivery Cesarean section	14/16 (87.5) 4/14 (28.6) 10/14 (71.4)
Gestational age at delivery (*week*)	35.5 (32–37)
Neonatal weight *(gram)*	1456.5 (1050–2135)
Sex Female Male	11/14 (78.6) 3/14 (21.4)

Table 2 shows the main features of the 16 babies followed in our Center, while Table 3 reports specific system-abnormalities detected at prenatal ultrasounds.

**Table 2:** Main features of the 16 cases with prenatal diagnosis of Trisomy 18

Case	GA at diagnosis (week)	Invasive procedures	Ultrasound findings	Outcome	Surgery
1 (R.A.)	13	CVS	Heart, cerebral, structural abnormalities and CAKUT	CS, 37 weeks, female, BW 1550 g, infant death	Surgical repair of the interventricular and interatrial defect and gastrostomy tube placement
2 (V.R.M.)	19	Amniocentesis	Heart, cerebral and structural abnormalities	VB, 37 weeks, female, alive at home (6 y.o.)	Cardiac surgery and gastrostomy tube placement
3	15	Cystocentesis and paracentesis	Heart and and structural abnormalities and CAKUT	IUFD	NA
4 (V.M.M.)	21	Amniocentesis	Heart, cerebral, structural and respiratory abnormalities	CS, 37 weeks, female (twin pregnancy), BW 1145 g, neonatal death	NA
5 (R.E.)	NA	Amnioreduction	Heart, cerebral and gastrointestinal abnormalities**,** IUGR	CS, 34 weeks, female, BW 1165 g, neonatal death	NA
6 (T.L.)	20	Amniocentesis	Heart and cerebral abnormalities, IUGR	CS, 35 weeks, female, BW 1500 g, neonatal death	NA
7 (L.Z.)	18	Amniocentesis	Gastrointestinal and renal abnormalities**,** IUGR	CS, 33 weeks, female, BW 1050 g, neonatal death	NA
8 (M.C.)	14	Amniocentesis	Heart, cerebral and gastrointestinal abnormalities**,** IUGR	VB, 34 weeks, female, BW 1435 g, infant death	Emergency surgery for gastric perforation and gastrostomy tube placement
9 (A.P.A.)	31	Amniocentesis	Heart, cerebral, structural and renal abnormalities, IUGR	VB, 34 weeks, female, BW 1550 g, neonatal death	NA
10 (B.G.M.)	14	CVS	Heart, cerebral, gastrointestinal and genital abnormalities**,** IUGR	CS, 37 weeks, female, BW 2135 g, neonatal death	NA
11 (C.G.)	NA	NA	Heart, cerebral, structural, gastrointestinal abnormalities, IUGR	CS, 37 weeks, male (twin pregnancy), BW 1660 g, neonatal death	NA
12 (S.E.)	17	Amniocentesis	Heart, cerebral, structural, genital abnormalities**,** IUGR	CS, 36 weeks, male, BW 1500 g, neonatal death	NA
13 (M.F.)	17	Amniocentesis	Heart, respiratory, structural and gastrointestinal abnormalities	CS, 37 weeks, female, BW 1960 g, neonatal death	NA
14	NA	NA	Heart, respiratory and structural abnormalities**,** IUGR	IVG	NA
15 (R.M.)	28	Amniocentesis	Heart, respiratory, structural and cerebral abnormalities	CS, 32 weeks, female, BW 1130 g, neonatal death	NA
16 (D.N.I.)	13	CVS and amnioreduction	Heart, structural and gastrointestinal abnormalities, IUGR	VB, 35 weeks, female, BW 1215 g, neonatal death	NA

*NA (not applicable); CS (cesarean section); VB (vaginal birth); CVS (chorionic villous sampling); BW (birth weight); IUGR (intrauterine growth restriction).* In case 11, diagnosis was performed after birth through genetic analysis

**Table 3:** Ultrasound abnormalities in T18 fetuses (both liveborn and non-liveborn)

	*All cases n* = *16*
*Cardiac*	
Ventricular septal defect	10 (62.5%)
AVC	3 (18.7%)
Tetralogy of Fallot	2 (12.5%)
Aortic coarctation	2 (12.5%)
Patent ductus arteriosus	1 (6.2%)
ARSA	1 (6.2%)
Polyvalvular disease	1 (6.2%)
Hypoplastic right heart	1 (6.2%)
Hypoplastic left heart	1 (6.2%)
Univentricular heart	1 (6.2%)
Pericardial effusion	1 (6.2%)
*Cerebral*	
Enlarged cisterna magna	5 (31,2%)
Dandy-Walker Syndrome	3 (18.7%)
Agenesis of the corpus callosum	2 (12.5%)
Ventriculomegaly	2 (12.5%)
Choroid plexus cysts	2 (12.5%)
Microcephaly	1 (6.2%)
Chiari malformation type 2	1 (6.2%)
*Genitourinary tract*	
Bilateral cystic renal dysplasia	1 (6.2%)
Bilateral renal hypotrophy with megacystis	1 (6.2%)
Duplex renal collecting system	1 (6.2%)
Hypospadias	1 (6.2%)
*Structural*	
Positional foot deformities	7 (43.7%)
Arthrogryposis	3 (18.7%)
Overriding fingers	1 (6.2%)
Clenched hands	1 (6.2%)
Bilateral forearm aplasia	1 (6.2%)
*Gastrointestinal*	
Omphalocele	3 (18.7%)
Small/absent stomach	2 (12.5%)
Echogenic bowel	2 (12.5%)
*Respiratory*	
Diaphragmatic hernia	1 (6.2%)
Pleural effusion	1 (6.2%)

*AVC (atrioventricular canal); ARSA (aberrant right subclavian artery)*

The most common ultrasonographic findings were intrauterine growth restriction and interventricular septal defects both detected in 10 (62,5%) fetuses, polyhydramnios in 6 (37,5%) fetuses, enlarged cisterna magna in 4 (25%) fetuses, cerebellum hypoplasia in 3 fetuses (18,7%), renal abnormalities in 3 fetuses (18,7%), arthrogryposis in 3 cases (18,7%), omphalocele in 3 fetuses (18,7%), clubfoot in 3 cases (18,7%), agenesis/dysgenesis of the corpus callosum in 2 fetuses (12,5%), absence of the nasal bone in 2 fetuses (12,5%), single umbilical artery in 2 babies (12,5%) and choroid plexus cysts in 1 (6%) fetus.

Analyzing systems in depth, cardiac prenatal defects were found in 93.7% of cases, including ventricular septal defects (62.5%), atrioventricular canal (18.7%), tetralogy of Fallot (12.5%), aortic coarctation (12.5%), patent ductus arteriosus (6.2%), aberrant right subclavian artery (6.2%), polyvalvular disease (6.2%), pericardial effusion (6.2%), univentricular heart (6.2%), hypoplastic left heart (6.2%) and hypoplastic right heart (6.2%). Only one baby had no cardiac abnormalities prenatally. After birth, 5 babies had an echocardiogram performed, and 2 of them underwent corrective cardiac surgery.

Structural abnormalities were recorded in 71.4% of cases, mainly represented by lower extremity deformities and arthrogryposis (43.7% and 18.7%, respectively).

Respiratory defects were detected in 14.3% of the fetuses antenatally, while genitourinary defects interested almost half of our study population. Specifically, the main abnormalities concerning the genitourinary tract were a bilateral renal hypotrophy associated with megacystis in a baby who died in utero; a bilateral cystic renal dysplasia; a duplex renal collecting system and hypospadias.

Prenatal central nervous system defects affected almost all the babies (78.5%). The main abnormalities found were enlarged cisterna magna (31,2%), Dandy-Walker Syndrome (18.7%), agenesis of the corpus callosum (12.5%), ventriculomegaly (12.5%), choroid plexus cysts (12.5%), microcephaly (6.2%), and Chiari malformation type 2 (6.2%).

Survival rate was commonly less than 1 day (50%), however two girls, A.R. and M.V.R., survived over 4 years.

### Questionnaire

**Fig. 2:**
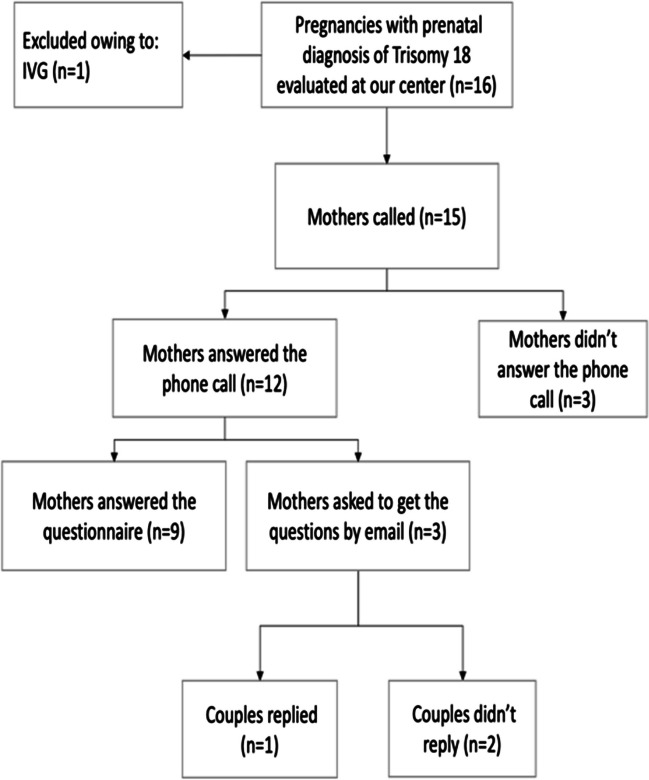
Flowchart summarizing couple’s participation to the survey

A 4-item questionnaire was administered to the couples enrolled. One couple was excluded as they chose to terminate the pregnancy. The remaining 15 couples were contacted by phone at the end of the data collection to answer the questionnaire. 12 couples answered the phone call while 3 couples didn’t. Among the 12 couples who responded, 3 of them requested to receive the questions by email, however only one replied (Fig. 2)*.* At the time of the survey, only one baby girl was still alive, at the age of 6. We created a new questionnaire tailored to the population we enrolled, based on questionnaires already used in the literature and on the main concerns we observed in couples during follow-up, such as the relationship with the partner and the possibility of future pregnancies. We intentionally chose open-ended questions to give couples the opportunity to express themselves and openly share their feelings.

Answers were analyzed and the main results were categorized as follows:

1. Reasons for continuing the pregnancy;
2. Couples’ joint decision;
3. Any second thoughts or regrets about going back;
4. Impact of the prenatal diagnosis on couples’ desire to have another baby.

### Reasons for continuing the pregnancy

Six couples (60%) made that decision for religious beliefs, other than that 5 couples (50%) stated that they didn’t feel right to make a choice about their baby’s life only because it was not the baby they expected, since its life had to be protected. One woman said she hoped that something could have been made to save her daughter. Also, 3 couples (21.4%) remarked that they had been trying to get pregnant for a long time so they didn’t feel right to interrupt the pregnancy.

### Couples' joint decision

Seven couples (70%) stated that both the parents agreed from the beginning. In 2 cases (20%), at the beginning the father was doubtful but then agreed. Finally, in 1 case (10%) the parents kept on disagreeing until the end.

### Any second thoughts

All the 10 couples (100%) who answered the questionnaire stated that they are happy about the choice they made and that they would do it again.

### Impact of the prenatal diagnosis on couples’ desire to have another pregnancy

Seven couples (70%) did not have other pregnancies subsequently but 2 of them are trying. Three couples (30%) had at least another pregnancy, however in one case it resulted in a miscarriage, while another couple is expecting the second child.

## Discussion

As the presented cases show, the prognosis of trisomy 18 is heterogeneous and it should not be blindly considered a lethal condition: these children can be born alive and enrich their families. However, the prognosis is generally poor and clinicians must be aware of the possible causes of death and morbidity associated, to provide these babies with the best care. In our cases, the most common cause of death was cardiopulmonary failure.

As reported in literature, the postnatal survival rate is higher for females, as for the prenatal period [2, 5, 13], and our observations were consistent with this data: we had 11 liveborn females and 3 liveborn males (78.6% vs 21.4%, respectively). All the males died within few hours, while females had the best survival rates.

The weight at birth is usually low, as extensively reported in literature [14, 15]. The mean neonatal weight of our cases was 1456,5 gr for a mean gestational age at birth of 35,5 weeks.

Feeding difficulties are common: most children either require a nasogastric tube or are gastrostomy dependent. In our study 3 (21.4%) children underwent surgical treatments after the delivery and they all required a gastrostomy placement since they didn’t manage to be fed spontaneously.

Considering congenital defects, cardiovascular anomalies are the most common feature observed: about 45–95% of patients diagnosed with trisomy 18 have congenital structural heart defects. In our series cardiac prenatal defects were found in 93.7% of cases, with ventricular septal defects being the most reported (62.5%).

Respiratory complications are another leading cause of mortality. Most children require some kind of respiratory support like oxygen therapy, non-invasive ventilation or invasive mechanical ventilation [2, 5, 6, 8, 16]. In our series we reported a 14.3% rate of respiratory defects, in line with current literature.

Limbs are involved in about 28% of cases [8] and, as a result, few patients are able to walk autonomously and physical therapy plays a fundamental role. Among the children we followed, 43.7% of them showed positional foot deformities, either prenatally or at birth, 6.2% showed overriding fingers, clenched hands and bilateral forearm aplasia. Also, arthrogryposis is common and 18.7% of the patients we followed showed it.

Genitourinary tract is often involved, with about 18% of children showing some form of abnormality [8]. In our population, we observed 43% rate of genitourinary tract abnormalities during pre and post-natal life, showing a higher rate compared to the literature.

Central nervous system abnormalities are also common: we found enlarged cisterna magna (31,2%), Dandy-Walker Syndrome (18.7%), agenesis of the corpus callosum (12.5%), ventriculomegaly (12.5%), choroid plexus cysts (12.5%), microcrania and microcephaly (6.2%), and Chiari malformation type 2 (6.2%), similarly to what has already been reported in the literature.

Moreover, trisomy 18 patients have an increased risk of developing specific neoplasia such as Wilms tumor, hepatoblastoma and Hodgkin disease. A.R. developed a suspected hepatoblastoma at the age of one.

Finally, a significant developmental impairment is present, with a psychomotor and intellectual disability. Both A.R. and M.V.R., the babies who survived the most in our study, had a psychomotor disability.

Many couples nowadays decide to continue the pregnancy and give birth. The prognosis is heterogeneous and depends on the amount and the type of malformations present and on the degree of medical interventions provided at birth. Some children manage to survive for several hours, days or months and in some cases, years, spending time with their families and enriching them. In 2014 Guon et al. analyzed the experience of parents whose children were diagnosed with trisomy 13 or 18, who decided to carry the pregnancy till term. About 35% of the couples had access to perinatal palliative care and most of them found it helpful: the study showed that the choice of carrying the pregnancy on had a positive effect on the majority of the couples and if they would have another pregnancy with trisomy 13 or 18 they would not abort in 91% of cases [11].

The series by Kosho et al. represents the largest group of parents who were asked to express their feelings about pregnancies with Trisomy 18 specifically. The main findings showed that parents appeared to be positive about caring for their children, although the difficulty to take care for physical condition and home medical care of their babies [15].

In a series by Janvier et al. parents of fetuses affected by Trisomy 18 and 13 were asked about the pregnancies’ impact on their quality of life. Almost all couples reported that their children enriched their life and had a positive effect on the relationship between mother and father. The most common negative comment was the feeling that healthcare providers didn’t see their baby as a unique and valuable human being [17].

Our study shows that the parents of children with Trisomy 18 approached the pregnancy process with a positive attitude, despite the difficulty in accepting the diagnosis and the resulting worries about the future. The concordance among parents in this important choice was very high (93%), and the total number of parents were happy with their choice to carry the pregnancy to term, supported by religious or purely ethical reasons.

None of the couples mentioned anxiety as a feeling during the journey, once they had taken the decision to accept the baby with his condition and carry the pregnancy to term. However, two couples stated they felt anxious at the time of the diagnosis due to the pressure to interrupt the pregnancy that came from the medical setting, but after meeting with our Perinatal Hospice team and fully accepting the situation, they all stated they were serene.

As strengths of our study, we can state that we reported a single center case-series with specific data on ultrasonographic, pediatric and obstetric follow-up; moreover, it is one of the longest follow-up studies with data on Trisomy 18 long-living babies and caregivers. The limitations of this study include the small number of cases analyzed, the retrospective nature of data collection and the use of a questionnaire not validated by the literature.

## Conclusions

Each patient has a different prognosis and the outcome can be varied, that is why the prenatal counselling is challenging for clinicians.

Trisomy 18 should not be considered merely a lethal condition: prolonged survival is possible, and these children can get to meet their parents and enrich their families. Nowadays many couples carry the pregnancy on, that is why healthcare providers must be aware of this syndrome and give families the support they need. The rising culture of the Perinatal Hospice is a response to the suffering of families struggling with difficult diagnosis who need a support and want their baby seen as unique and valuable. The methodology of the Perinatal Hospice, combining and interdisciplinary and multidisciplinary approach, implements a shared medicine between the family and the physician. This approach makes the couple perceive a sense of dignity in their choice and consider the baby separately from his illness.

In conclusion, Perinatal Hospice is a reality in which families can feel supported and accompanied along this difficult journey and a way to honor a baby’s life since it has an intrinsic value, no matter how fleeting and “imperfect”.

## Data Availability

No datasets were generated or analysed during the current study.
